# Exploring the role of quantitative susceptibility mapping in assessing brain iron deposition in hemodialysis patients

**DOI:** 10.1186/s13244-025-02197-x

**Published:** 2026-02-16

**Authors:** GuoLi Ren, QingQing Nie, Daliang Liu, Bo Wang, Xiao Gao, XueHuan Liu, Hao Wang, Jun Liu

**Affiliations:** 1 Tianjin Medical University, Tianjin Fourth Central Hospital, Tianjin, China; 2 Liaocheng People’s Hospital, Liaocheng, China; 3 Liaocheng Hospital of Traditional Chinese Medicine, Liaocheng, China; 4 Tianjin Union Medical Center, Tianjin, China

**Keywords:** End-stage renal disease, Hemodialysis, Magnetic resonance imaging, Iron deposition, Cognitive impairment

## Abstract

**Abstract:**

Patients with end-stage renal disease (ESRD) develop brain iron deposition due to iron metabolism disorders induced by long-term hemodialysis. This abnormal iron accumulation accelerates cognitive impairment (CI) and neurodegenerative pathologies. Quantitative susceptibility mapping (QSM), a technique capable of precisely quantifying magnetic susceptibility, provides a novel perspective for the noninvasive and dynamic monitoring of cerebral iron distribution. Monitoring brain iron deposition using QSM facilitates the development of individualized clinical treatment strategies. This review systematically examines the application of QSM in studying brain iron deposition in hemodialysis patients, with a focus on analyzing the dynamic patterns of iron deposition pre- and post-dialysis and during follow-up periods. It further explores the relationship between QSM findings and iron metabolism dysregulation, blood-brain barrier (BBB) injury, and oxidative stress. Additionally, the predictive value of QSM for clinical neurological functional prognosis following iron chelation therapy is discussed.

**Critical relevance statement:**

QSM studies on cerebral iron deposition in hemodialysis patients require further monitoring of its spatial-temporal dynamics and changes after iron chelation. Future research should focus on technical standardization, longitudinal tracking, and treatment response to establish a precision neuroimaging-guided framework.

**Key Points:**

- This review exploration is warranted to monitor the spatial distribution and dynamic changes of brain iron deposition in this population.
- The relationships between QSM findings and iron metabolism dysregulation, blood-brain barrier injury, and oxidative stress are explored.
- This review focuses on issues in the fields of technology standardization, longitudinal monitoring, and treatment responsiveness.

**Graphical Abstract:**

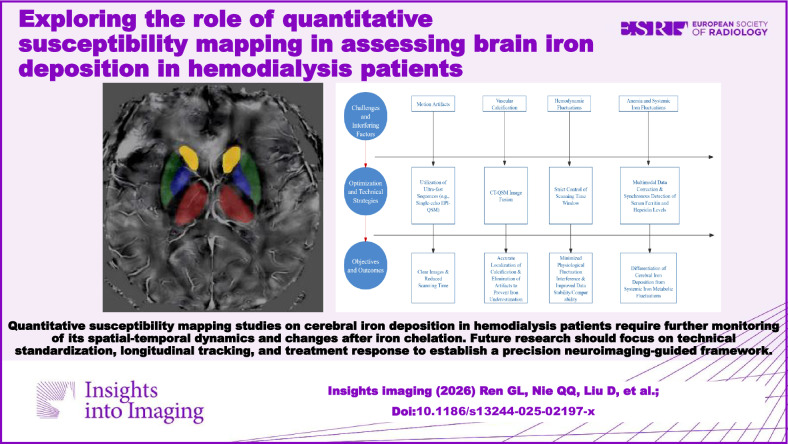

## Introduction

Patients with end-stage renal disease (ESRD) undergoing maintenance hemodialysis frequently experience progressive brain injury, manifesting as ccomplications including cognitive impairment (CI), movement abnormalities, and accelerated cerebral atrophy [1–3]. Among the underlying pathological mechanisms driving such neurodegeneration, dysregulation of brain iron homeostasis is a core factor [4]. Conventional imaging techniques (e.g., T2*-weighted imaging, R2* mapping from gradient recalled echo sequences) are limited in achieving precise quantification of cerebral iron concentration due to interference from susceptibility artifacts [5, 6]. In recent years, quantitative susceptibility mapping (QSM) has been widely applied to quantify the spatial distribution of magnetic susceptibility in brain tissue [6, 7]. The magnetic susceptibility values (MSV) derived from this method have been demonstrated to correlate significantly with histochemical iron concentrations [8], thereby providing an objective tool for quantitative imaging analysis of brain iron in clinical settings [9].

**Fig. 1:**
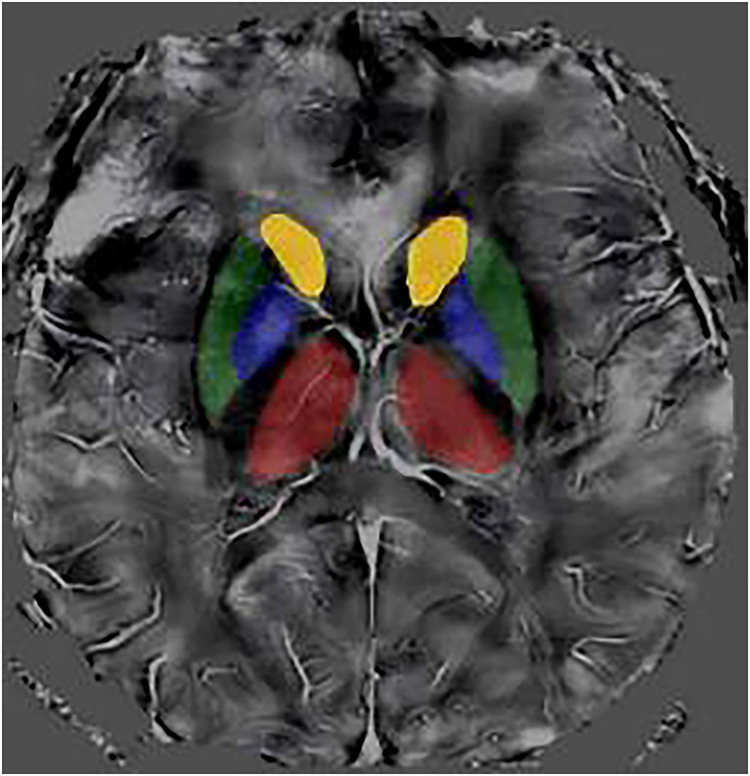
Representative quantitative susceptibility mapping (QSM) of deep gray matter structures. The ROIs are shown in four distinct colors: yellow for bilateral caudate nuclei, green for the bilateral putamen, blue for the bilateral pallidum, and red for the bilateral thalamus

Although cross-sectional studies have revealed increased iron deposition in deep gray matter nuclei (e.g., head of the caudate nucleus, putamen are as shown in Fig. 1) of dialysis patients, a systematic understanding remains lacking regarding the relationship between dynamically monitored changes in brain iron deposition and blood-brain barrier injury, as well as the specific impacts of dialysis sessions and long-term iron accumulation. Key unresolved questions include how spatial patterns of iron deposition can explain specific neurological symptoms and the potential of these patterns to serve as biomarkers for treatment response.

This review focuses on the unique value of QSM for the dynamic monitoring of brain iron deposition in hemodialysis patients. It aims to: (1) synthesize multi-temporal scale evidence (pre-dialysis baseline → post-dialysis treatment (> 24 h after dialysis) → long-term follow-up) to map the evolution of iron deposition; (2) establish dose-response models linking the spatial distribution of iron to impairments in cognitive and motor domains; and (3) guide clinical targeted iron chelation therapy. Ultimately, this work seeks to provide imaging biomarkers for personalized clinical management.

## QSM imaging technology adapted for dialysis scenarios

### QSM imaging technology

QSM quantifies local tissue magnetic susceptibility by post-processing MRI data—including phase unwrapping and background field removal. Morphology-enabled dipole inversion (MEDI) is a computational method for reconstructing magnetic susceptibility distributions from field data [6, 10–12]. QSM generates susceptibility maps where negative values indicate diamagnetic properties and positive values indicate paramagnetic properties [12]. After QSM map reconstruction, data application strategies must be defined: (1) Region-of-interest (ROI) analysis: widely used for anatomical structures (e.g., cortex, white matter, deep gray matter) and pathology-specific regions. While reducing noise, averaging positive/negative values may obscure physiological information [13]. (2) Voxel-wise analysis: better preserves diamagnetic/paramagnetic contrasts but is noise-sensitive. Recent studies advocate independent analysis of positive/negative susceptibility values [14–17]. But reference region selection (cerebrospinal fluid, white matter, whole brain) affects ROI susceptibility standard deviations.

Compared to other susceptibility-weighted sequences such as T2*-weighted imaging, QSM offers superior quantification, distinguishes local from non-local tissue properties, exhibits higher sensitivity to susceptibility effects, and is less affected by imaging parameters and orientation [18–20]. In brain research, QSM has been utilized to analyze susceptibility alterations caused by changes in iron, calcium, myelin, or lipid content across various neurological disorders [18–20]. Iron holds particular significance in neurodegenerative and aging research due to its quantum spin properties. Regardless of the specific neurodegenerative pathology, iron contributes to cell death and neuronal loss by triggering inflammatory responses—including induction of oxidative damage, reactive oxygen species generation, and iron-dependent cell death (ferroptosis) [21–23]. Furthermore, iron exacerbates neurodegeneration by interacting with aggregates of pathogenic proteins such as amyloid-β plaques, tau neurofibrillary tangles, and α-synuclein [24–26]. However, study results indicate that the choice of algorithm significantly influences the ability of QSM processing to detect physiological changes in the brain. Thus, the configuration of the data processing pipeline warrants careful consideration [27]. Variations in QSM data acquisition, processing, and analytical approaches may impact research outcomes and their correlations with other imaging biomarkers or clinical metrics.

### QSM imaging optimization strategies

**Fig. 2:**
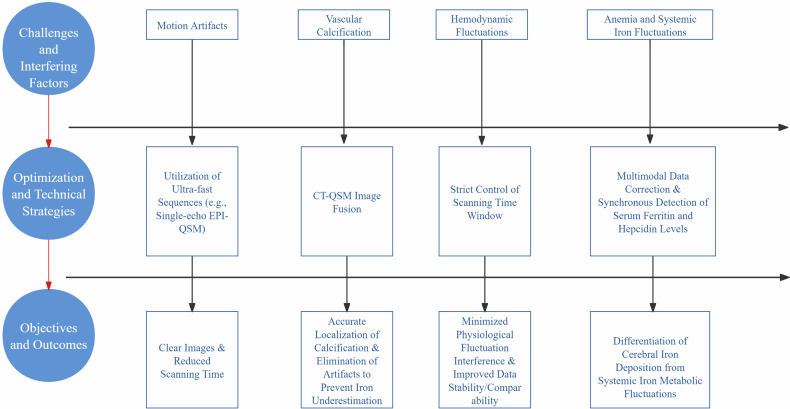
Flowchart of QSM imaging optimization strategies

This section systematically elaborates on the major challenges in QSM imaging for hemodialysis patients and the corresponding optimization strategies (Fig. 2). For hemodialysis patients, motion artifacts from limb movement or positional adjustments during scanning can be mitigated using ultrafast sequences (e.g., single-echo EPI-QSM), reducing scan time while ensuring image clarity [28]. Vascular calcifications (diamagnetic)—prevalent in dialysis patients, particularly in the basal ganglia—counterbalance iron deposition (paramagnetic) signals. This necessitates CT-QSM image fusion to localize calcified regions and generate susceptibility masks for artifact exclusion, preventing iron underestimation [29]. Hemodialysis induces hemodynamic fluctuations from circulatory pressure changes (e.g., electrolyte/volume shifts), causing transient hypotension, cerebral hypoperfusion, arterial hypoxemia, and recurrent ischemia [30–32]. Strictly controlled scanning time windows are required to minimize these confounders. Anemia—common in chronic kidney disease and exacerbated by hemodialysis—affects cerebral blood flow (CBF) quantification. Individual variations in blood longitudinal relaxation time (T1) must be accounted for [33]. Concurrent measurement of serum ferritin (SF) and hepcidin levels is essential to disambiguate true brain iron accumulation from systemic iron metabolism fluctuations. Key challenges-including motion artifacts, vascular calcification, hemodynamic fluctuations, and anemia-can be mitigated to a significant extent through targeted methods such as ultrafast sequences, image fusion, controlled scanning time windows, and multimodal data correction.

### Reproducibility of QSM across magnetic field strengths

The growing importance of QSM in medical research necessitates a rigorous assessment of its reproducibility. A study comparing QSM reproducibility and consistency between 1.5 T and 3 T field strengths demonstrated that bandwidth adjustment optimizes the contrast-to-noise ratio (CNR) at 1.5 T. MEDI-based QSM reconstruction maintained excellent reproducibility and consistency across both field strengths (1.5 T/3 T) and bandwidth variations [34]. While advanced reconstruction algorithms, such as MEDI, can partially bridge the disparities introduced by different field strengths and ensure data reliability in multi-center studies, they cannot entirely overcome the inherent physical differences. Therefore, field strength remains a critical variable that must be controlled and reported in experimental design. QSM measurements exhibit strong dependence on echo time (TE): identical tissues scanned on the same scanner show different apparent frequency shifts depending on TE selection [35]. In addition to scanning parameters (e.g., TE), physiological variations among subjects, particularly in longitudinal relaxation time (T1), also constitute a key variable affecting the quantitative accuracy of QSM, especially in the specific population of hemodialysis patients. Comparative evaluation of 3-T and 7-T systems revealed that using TEs ≈2.6 times longer than those at 7 T enables highly reproducible whole-brain susceptibility mapping across scanners. This parameter optimization establishes QSM as a robust technique for longitudinal follow-up in clinical and multi-site studies [36]. In QSM reconstruction pipelines based on susceptibility-weighted imaging or those incorporating T1-weighted sequences, variations in T1 relaxation times directly influence tissue signal intensity. This intensity variation can be erroneously interpreted as differences in magnetic susceptibility. For instance, in dialysis patients, factors such as anemia and edema can lead to prolonged T1 values in brain tissue. If uncorrected, this may introduce a systematic bias in the quantification of brain iron [34]. Consequently, for studies involving hemodialysis patients, strictly controlling scanning parameters is necessary but insufficient. It is also crucial to employ QSM sequences that are less sensitive to T1 variations or to perform data correction through multimodal imaging, such as by simultaneously acquiring T1 maps. In conclusion, reproducibility assessments must account for field strength differences and scanning parameter variations and are more profoundly influenced by the individual physiological status of the subjects.

### Heterogeneity of iron deposition

The limited diagnostic precision of QSM-based tools primarily stems from conventional susceptibility measurements failing to capture iron deposition heterogeneity. Liu et al [37] proposed the high-susceptibility regional analysis and demonstrated its superior sensitivity over whole-structure analysis. Subsequent region-based studies confirm that regional iron load assessment provides critical insights into iron distribution patterns linked to CI and mood disorders [38]. Wang et al [39] measured magnetic susceptibility values in individual basal ganglia nuclei of chronic kidney disease (CKD) patients and performed habitat analysis based on radiomic features derived from QSM images of this region. This approach successfully constructed a model for accurately diagnosing CKD-associated cognitive impairment. Simple averaging of susceptibility values across basal ganglia regions fails to capture this heterogeneity. Such oversimplification explains the suboptimal diagnostic performance of regional susceptibility measurements in CKD-related CI [39]. Iron deposition heterogeneity may hold the key to diagnosing and treating CKD-associated CI, though this hypothesis requires further validation.

## Dynamic mapping of brain iron deposition in hemodialysis patients

### Baseline factors influencing iron deposition

Brain iron deposition in hemodialysis patients involves a multifactorial pathophysiological process. Key drivers identified include: (1) Renal anemia management: routine iron supplementation for hematocrit/hemoglobin maintenance may cause systemic iron overload, facilitating cerebral iron entry [40]. (2) Cerebrovascular insults: hemodialysis-induced small vessel diseases (white matter hyperintensities, stroke, microbleeds) promote pathological iron accumulation [41–43]. (3) Accelerated encephalatrophy: pathological gray matter nucleus atrophy correlates with increased iron deposition and is more severe in patients versus controls [2, 38]. In addition to the above three influencing factors, the influence of dialysis duration and age [44] should also be considered.

### Dynamic changes of brain iron deposition caused by a single dialysis

Hemodialysis itself may not directly cause iron deposition [45], however, acute hemodynamic shifts during treatment include blood pressure volatility: ≥ 20 mmHg systolic pressure (SBP) reductions in 25% of patients, often exceeding this threshold [46, 47], and transient perfusion alterations potentially contributing to acute confusional states, though cognitive function peaks 24 h post-dialysis [48]. Blood pressure and cognition correlation remains controversial [30]. While hemodynamic parameters typically normalize post-dialysis, recurrent sessions may cause permanent cerebrovascular dysfunction, exacerbating structural/functional brain abnormalities. Given the established cerebral blood flow-iron deposition linkage [4], further validation is needed regarding associations between iron deposition and clinical biomarkers (e.g., serum ferritin (SF) and dialysis prescriptions (duration, blood flow rate, dialyzer type)).

### Long-term cumulative effects (≥ 1-year follow-up)

Studies have shown that patients receiving long-term hemodialysis develop disorders of iron metabolism, with tissue iron overload observed in the spleen, pancreas, and liver [40]. Furthermore, increased iron deposition is evident in the deep gray matter nuclei (including the head of the caudate nucleus, putamen, substantia nigra, red nucleus, and dentate nucleus) of dialysis patients. Iron deposition in the head of the caudate nucleus correlates with CI, as the caudate nucleus is a key component of the anterior striatal circuit and plays a crucial role in neurocognitive function [2]. However, this study employed a cross-sectional design, and the influence of individual variations may introduce bias into the assessment of iron content changes. A subsequent prospective longitudinal study confirmed that iron deposition in the deep gray matter nuclei of hemodialysis patients progressively worsens over approximately 2 years and may serve as a risk factor for neurocognitive CI, with abnormal creatinine levels and disturbances in calcium-phosphorus metabolism identified as independent risk factors for abnormal iron deposition [45]. This study also revealed that the process of iron deposition in the thalamus and pulvinar is relatively slower and exhibits heterogeneity within these nuclei, with iron content changes being less sensitive compared to other brain nuclei [45]. Nevertheless, this research could not definitively delineate the specific manifestations of cognitive impairment caused by abnormal brain iron deposition. These findings again highlight the heterogeneity of brain iron deposition. Whether the differences in magnetic susceptibility within the deep gray matter nuclei of long-term hemodialysis patients are associated with brain injury and clinical symptoms warrants further investigation.

## Causal links between spatial patterns of iron deposition and neurological symptoms

**Fig. 3:**
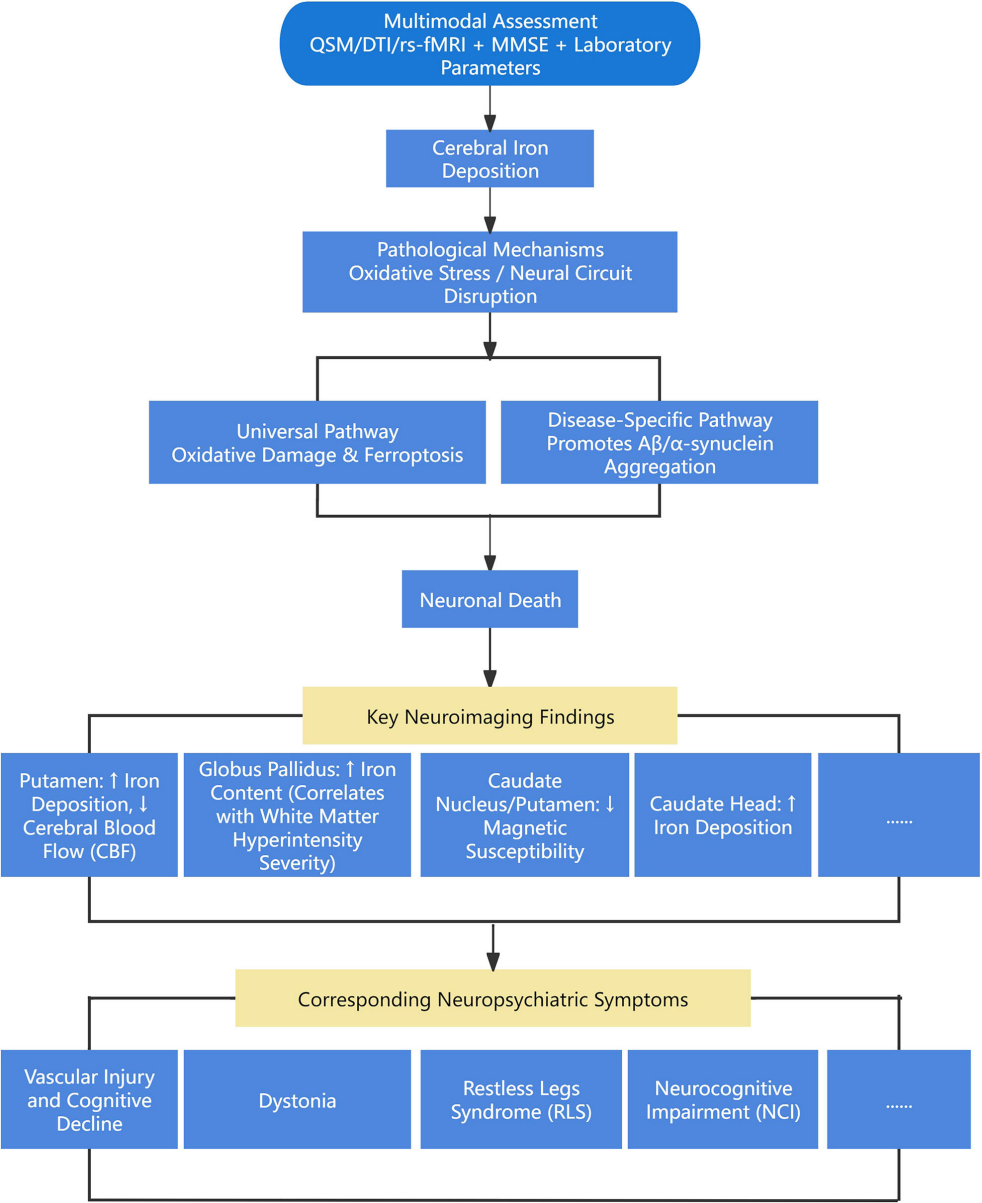
Schematic diagram illustrating the pathogenic role of cerebral iron deposition in neuropsychiatric complications

Spatial distributions of abnormal iron deposition exhibit causal relationships with specific neurological symptoms through mechanisms involving oxidative stress damage, disruption of neural circuits, and activation of molecular pathways. Evidence confirms that brain iron deposition in ESRD contributes to multiple neuropsychiatric complications, with CI being predominant and potentially progressing to dementia. CI affects 30%–60% of ESRD patients, manifesting as multidomain dysfunction encompassing global cognition, memory, executive function, and attention [49]. In ESRD patients with CI, aberrant functional connectivity (FC) is observed between brain regions responsible for distinct cognitive domains: decreased FC reflects impaired functional integration, while increased FC indicates compensatory neural reorganization. These alterations primarily involve major networks, including the default mode network (DMN), salience network, and executive control network [50]. Recent resting-state functional magnetic resonance imaging (rs-fMRI) studies reveal abnormalities in the amplitude of low-frequency fluctuations (ALFF) and regional homogeneity within DMN regions of CKD patients [51, 52]. Liu et al [53] demonstrated CKD-specific CBF increases predominantly in DMN regions, with precuneus CBF changes significantly correlating with executive dysfunction. In maintenance hemodialysis patients, elevated putamen iron deposition coincides with reduced CBF, suggesting vascular injury and dysfunction as central drivers of altered perfusion and iron dysmetabolism [54]. Susceptibility-ALFF mapping shows promise as an early biomarker of cognitive decline in CKD, providing mechanistic insights into associated neuropathology [55]. Independent associations exist between pallidal iron content and white matter hyperintensities severity [41]—where pallidal lesions correlate with dystonia—while reduced magnetic susceptibility in the caudate nucleus and putamen links to restless legs syndrome [56, 57]. Caudate head iron deposition further associates with neurocognitive impairment [2, 38]. Integrating diffusion tensor imaging with QSM enhances spatial multimodal correlations, improving precision in localizing iron deposition and quantifying white matter injury. The complex interplay between the spatial distribution of cerebral iron deposition, its functional consequences, and the ensuing clinical symptoms is summarized in Fig. 3. Nevertheless, studies comparing or combining these techniques for ESRD diagnosis and severity assessment remain scarce.

QSM-based monitoring of spatial-temporal iron dynamics advances understanding of iron-mediated complications in ESRD. Iron contributes to neuropathology through two principal mechanisms (Fig. 3) [21, 25]: (1) A universal pathway involving reactive oxygen species (ROS)-mediated oxidative damage and ferroptosis (an iron-dependent cell death mechanism) operative across diseased and healthy states. (2) A disease-specific pathway whereby iron interacts with pathogenic protein aggregates—including β-amyloid (Aβ) plaques, α-synuclein, and tau—promoting their production and aggregation. Iron incorporation into these structures enhances their oxidative capacity, ultimately driving neuronal death [25]. Diagnosing iron-mediated neuropathology requires integration with clinical assessments (e.g., Mini-Mental State Examination (MMSE)) and laboratory parameters (e.g., serum phosphorus).

## Potential for treatment-responsive biomarkers

### Iron therapy in dialysis patients

The treatment of anemia has become a primary concern for end-stage renal disease (ESRD) patients on dialysis [58]. International KDIGO (Kidney Disease: Improving Global Outcomes) guidelines recommend intravenous (IV) iron administration when serum ferritin (SF) is < 500 µg/L and transferrin saturation (TSAT) is < 30%, but advise against routine iron use in patients with SF > 500 µg/L or TSAT > 30% [59, 60]. The guidelines also suggest considering IV iron for patients with SF > 500 µg/L who exhibit low hemoglobin levels despite high-dose erythropoiesis-stimulating agent (ESA) therapy, or for those preferring to discontinue ESA treatment [59, 60]. Iron therapy should be discontinued when hemoglobin levels exceed 130 g/L or when SF exceeds 500 µg/L, and treatment should be continued for up to 6 months [61]. SF serves as a crucial indicator for assessing iron deficiency or overload, and elevated SF levels are frequently observed in hemodialysis patients [62]. Study results indicate that high SF is a risk factor for mortality in hemodialysis patients, and monitoring SF levels can aid in predicting prognosis [63]. Rostoker et al [64] reported that in a study of 119 dialysis patients receiving ESA and IV iron, 84% had concomitant iron overload, with MRI even showing hepatic iron loading reaching levels consistent with hemochromatosis in 30% of patients. Current evidence indicates that these conventional markers (SF, TSAT) often fail to accurately predict iron deficiency or overload status in hemodialysis patients. The team led by Barbieri developed a data-driven machine learning model—the Anemia Control Model—which integrates patient history, dose-response data, and demographic characteristics to provide individualized ESA dosing recommendations for hemodialysis patients, thereby supporting anemia management [65]. The model combines an artificial neural network with a dose-selection algorithm to achieve optimal dosing. Brain iron overload may lead to severe neurological disorders, underscoring the necessity of preventing iron overload. Routine screening mechanisms for iron overload should be established in dialysis patients, particularly in those receiving high cumulative doses of IV iron or undergoing long-term dialysis.

### Monitoring of iron chelator interventions

Balancing the need to improve hemoglobin levels with the prevention of iron overload presents a clinical dilemma. First, the amount of iron released from blood transfusions is typically higher than the actual dose received by the patient, yet accurately tracking this through real-world monitoring can be challenging. More importantly, complex interactions exist between the rate of iron intake from transfusions and the efficacy of chelators. These interactions may vary based on individual patient differences and disease characteristics, potentially leading to discrepancies between predicted and observed treatment outcomes [66]. Magnetic resonance imaging has become the established gold standard for monitoring in vivo iron levels due to its accuracy, reliability, reproducibility, good patient tolerance, and ability to track iron content changes across different organs, primarily detecting iron in the liver, heart, pancreas, and pituitary gland [66, 67]. However, the mechanisms and kinetics of iron uptake and clearance differ among bodily organs. Currently, studies utilizing QSM to monitor changes in iron deposition within the deep gray matter nuclei of ESRD patients following iron chelator intervention have not been documented. Existing research has confirmed a decrease in R2* values within the deep brain gray matter nuclei after deferiprone treatment for brain iron deposition [67]. This provides a basis for monitoring the dynamic changes in brain iron overload following iron chelator intervention. Implementing MRI monitoring in practice requires consideration, as it is a relatively expensive medical procedure, necessitating judicious use of this resource. Nevertheless, its monitoring cost is relatively low when compared to the expenses associated with iron chelating drugs and the costs incurred from iron-mediated complications.

Beyond conventional monitoring, emerging evidence indicates that QSM has become a vital tool for predicting neurodegenerative pathology. A longitudinal study investigating clinical severity over 3 years in 59 PD patients without dementia symptoms at baseline demonstrated that QSM may indicate cognitive functional deterioration months before the onset of overt cognitive impairment in PD patients [68]. Additional research has robustly established QSM’s significant capacity to predict the future rate of both cognitive and motor function decline [69]. Applying this finding to the ESRD patient population, serial QSM examinations hold promise for identifying individuals at the highest risk of transitioning from mild cognitive impairment to dementia, thereby enabling preventive interventions. Furthermore, QSM has direct application in guiding clinical decision-making for iron chelation therapy. While current guidelines rely on systemic parameters, the frequent discordance between systemic and cerebral iron levels underscores the necessity for developing brain-specific biomarkers. Prospective studies could utilize iron load thresholds in the caudate nucleus or putamen, as detected by QSM, as triggers for initiating or intensifying chelation therapy. Subsequent QSM scans would then provide an objective assessment of cerebral iron chelation efficacy, overcoming the limitations of the current paradigm, which relies solely on serum ferritin levels and clinical experience for dose adjustment. This targeted therapeutic strategy has the potential to maximize efficacy while minimizing patient exposure to potentially toxic medications.

## Challenges and future perspectives

Cerebrospinal fluid pulsation artifacts will interfere with the quantitative analysis of cortical regions. While cognitive function is often associated with cortical areas, the irregular morphology and poorly defined boundaries of these regions make the precise definition of cortical ROIs challenging. Furthermore, transient susceptibility interference in QSM values caused by intravenous iron infusion can confound the long-term detection of brain iron deposition. Performing QSM scans within 48 h post-iron infusion should be avoided; prioritizing low-susceptibility iron formulations and conducting longitudinal follow-up with multiple scans can significantly enhance the reliability of QSM for detecting brain iron deposition in hemodialysis patients.

The integration of artificial intelligence into the clinical management of chronic kidney disease holds significant potential to enhance early screening, enable accurate prediction of treatment outcomes, and improve patient prognosis. To achieve this, collaboration among healthcare institutions, research teams, regulatory bodies, and industry partners is essential for establishing standardized protocols that comply with regulatory requirements, ensuring patient safety and minimizing medical risks [70]. From a methodological perspective, deep learning (DL)-QSM has been shown to facilitate quantitative susceptibility mapping of deep brain nuclei [71]. In another study, Kalmrowski et al employed a random forest model incorporating patient age, sex, and dynamic laboratory parameters to predict the risk of kidney failure within 6 and 12 months in patients with advanced CKD [72]. The model identified 88% of unplanned dialysis cases at 6 months, maintaining an accuracy of 87% at 12 months, with external validation consistently achieving 87% accuracy at both time points. Such early-warning systems support clinical decision-making, allowing timely intervention and facilitating a smoother transition to renal replacement therapy.

A key direction for future research lies in applying multimodal AI frameworks to integrate diverse datasets. By combining multidimensional data from QSM, fMRI, and PET, multimodal machine learning models such as graph neural networks can be trained to develop superior predictive models. Combining quantitative susceptibility mapping (QSM) with resting-state functional MRI (fMRI) may reveal critical relationships between local iron deposition and abnormalities in functional connectivity networks, such as the default mode network [55]. Correlating QSM findings with positron emission tomography offers significant research value. Aβ-PET can uncover iron-Aβ co-localization, helping to elucidate Alzheimer’s disease-like mechanisms in end-stage renal disease (ESRD)-related cognitive decline. Similarly, tau-PET or neuroinflammation PET (e.g., TSPO-PET) can clarify the dual contributions of tau pathology and glial activation to disease phenotypes, moving beyond a sole focus on iron [9]. These models could not only track iron dynamics but also predict trajectories of cognitive decline and identify neurodegenerative subtypes enriched in ESRD, ultimately supporting precision medicine.

The clinical translation of QSM depends on two key factors: establishing dynamic multi-timepoint monitoring protocols and defining QSM-based standards for assessing responses to iron chelation therapy. By deeply integrating AI—especially through multimodal imaging paradigms—QSM has the potential to evolve from a standalone quantitative tool into an essential component of personalized treatment strategies for hemodialysis patients.

## Summary

Significant progress has been made in QSM imaging studies of brain iron deposition in hemodialysis patients. However, further research and exploration are warranted to monitor the spatial distribution and dynamic changes of brain iron deposition in this population, as well as the alterations in brain iron deposition following iron chelator intervention. QSM, as a noninvasive and highly sensitive MRI technique, provides a novel tool for analyzing the dynamic evolution of brain iron deposition in hemodialysis patients, investigating its underlying mechanisms, and evaluating therapeutic efficacy. Future research should deepen exploration in areas such as technical standardization, longitudinal monitoring, and treatment responsiveness, thereby contributing to the establishment of a precision neuroimaging-guided diagnosis and treatment framework.

## Abbreviations

CBF: Cerebral blood flow
CI: Cognitive impairment
CKD: Chronic kidney disease
DMN: Default mode network
ESA: Erythropoiesis-stimulating agent
ESRD: End-stage renal disease
FC: Functional connectivity
MEDI: Morphology-enabled dipole inversion
QSM: Quantitative susceptibility mapping
ROI: Region-of-interest
SF: Serum Ferritin
TSAT: Transferrin saturation
